# The American Medical Formulary, Based upon the United States and British Pharmacopeias

**Published:** 1850-07

**Authors:** 


Art. VIII.—*The American Medical Formulary, based upon the United States and British Pharmacopeias. Including also, numerous standard Formula, derived from American and European authorities. Together with the Medical Properties and uses of Medicines; Poisons, their Antidotes, Tests, etc. Designed for the Medical and Pharmaceutical Student.* By John J. Reese, M. D., Lecturer on Materia Medica and Therapeutics in the Philadelphia Medical Institute, &c. Philadelphia: Lindsay & Blakeston. 1850.

The old adage, "it never rains but it pours," may well be applied to the inundation of books of medical formularies, which has recently taken place. Within a very limited time, the American public has been blessed with Mayne's Dispensatory and Formulary, a new edition of Ellis's work, Griffith's Universal Formulary, and Reese's American Medical Formulary.

In our notice of Griffith's Universal Formulary, we expressed ourselves in very plain terms on the subject of Medical Formularies, and it is scarcely necessary to repeat now, what we said at that time. But we hope we shall not be understood as attempting to depreciate the labors of Dr. Reese in the work before us. He has prepared a good work, in this department of books, and one that can be relied on. In addition to the Pharmacopeia, he has some useful matter in an appendix, which though useful scarcely belongs to a Pharmacopeia. We allude to the articles on Dietetic Preparations, Poisons, Mineral Springs, &c.

This work belongs to the series, entitled: "*The Medical Practitioner's and Student's Library*," in the course of publication by Lindsay & Blakeston.

